# *Drosophila* Histone Deacetylase-3 Controls Imaginal Disc Size through Suppression of Apoptosis

**DOI:** 10.1371/journal.pgen.1000009

**Published:** 2008-02-29

**Authors:** Changqi C. Zhu, Douglas J. Bornemann, David Zhitomirsky, Ellen L. Miller, Michael B. O'Connor, Jeffrey A. Simon

**Affiliations:** 1 Department of Genetics, Cell Biology and Development, University of Minnesota, Minneapolis, Minnesota, United States of America; 2 Howard Hughes Medical Institute, Chevy Chase, Maryland, United States of America; Stowers Institute for Medical Research, United States of America

## Abstract

Histone deacetylases (HDACs) execute biological regulation through post-translational modification of chromatin and other cellular substrates. In humans, there are eleven HDACs, organized into three distinct subfamilies. This large number of HDACs raises questions about functional overlap and division of labor among paralogs. In vivo roles are simpler to address in *Drosophila*, where there are only five HDAC family members and only two are implicated in transcriptional control. Of these two, HDAC1 has been characterized genetically, but its most closely related paralog, HDAC3, has not. Here we describe the isolation and phenotypic characterization of *hdac3* mutations. We find that both *hdac3* and *hdac1* mutations are dominant suppressors of position effect variegation, suggesting functional overlap in heterochromatin regulation. However, all five *hdac3* loss-of-function alleles are recessive lethal during larval/pupal stages, indicating that HDAC3 is essential on its own for *Drosophila* development. The mutant larvae display small imaginal discs, which result from abnormally elevated levels of apoptosis. This cell death occurs as a cell-autonomous response to HDAC3 loss and is accompanied by increased expression of the pro-apoptotic gene, *hid*. In contrast, although HDAC1 mutants also display small imaginal discs, this appears to result from reduced proliferation rather than from elevated apoptosis. The connection between HDAC loss and apoptosis is important since HDAC inhibitors show anticancer activities in animal models through mechanisms involving apoptotic induction. However, the specific HDACs implicated in tumor cell killing have not been identified. Our results indicate that protein deacetylation by HDAC3 plays a key role in suppression of apoptosis in *Drosophila* imaginal tissue.

## Author Summary

Histone deacetylases (HDACs) are enzymes that reverse acetylation of protein substrates inside the cell. Like phosphorylation, acetylation/deacetylation can alter the biochemical properties of a protein target and thereby regulate its functions. Histones are a major target of certain HDACs. When histones become deacetylated, the biochemical properties of the local chromatin are altered, which can contribute to gene silencing. HDACs can also act upon protein substrates besides histones. There are multiple HDACs encoded in animal genomes, with eleven HDACs in humans. Thus, it becomes complicated to determine which individual HDACs exert which biological functions in vivo. To address this, we have isolated and studied mutations that specifically disrupt a single HDAC, HDAC3, in Drosophila. We find that a major function of HDAC3 is to prevent programmed cell death from occurring abnormally in certain fly tissues. This finding has implications for anticancer strategies since HDAC chemical inhibitors can reduce tumors in animal models through induction of cell death. Our study identifies HDAC3 as a single HDAC among many that can play a key role in control of cell death and suggests that this version of the enzyme should be further investigated for regulatory roles in tumor cell killing versus survival.

## Introduction

Histone deacetylases (HDACs) are members of an ancient enzyme family that reverses acetylation of protein substrates. The most well-characterized HDAC substrates are the N-terminal tails of the histones. Acetylation of histone tail lysines generally correlates with gene activity, whereas HDAC-sponsored removal of these tail modifications frequently accompanies gene silencing [1],[2]. Histone acetylation state can impact gene expression through recruitment of transcriptional regulatory complexes, such as the SWI/SNF remodelling complex [3],[4]. Changes in charge density resulting from histone acetylation/deacetylation may also affect packaging of nucleosome arrays into higher-order arrangements that can impact transcription rates. A major HDAC regulatory function, then, is to promote gene silencing.

The histone deacetylase HDAC1 has been the most throughly studied HDAC at the biochemical and functional levels. Extensive analysis of HDAC1 in yeast, also known as RPD3, indicates that it can deacetylate all four core histones, that it targets hundreds of genes around the genome, and confirms its major role as a direct transcriptional repressor [1], [5]–[8]. Biochemical studies show that HDAC1 is typically assembled into nuclear complexes, such as the SIN3 and NURD complexes [2], [9]–[11]. These co-repressor complexes are recruited to target genes through interactions with DNA-binding proteins. A prime example is provided by nuclear hormone receptors such as thyroid hormone receptor; the unliganded receptor recognizes target genes through its zinc finger DNA-binding domain, and it recruits a SIN3/NCoR/HDAC1 complex, which deacetylates target chromatin and leads to gene silencing [12].

As a consequence of their roles with many co-repressors, HDACs have widespread function around the genome and they participate in many gene regulatory systems. In addition to steroid hormone receptor control in vertebrates and invertebrates [12],[13], HDACs also function in the TGF-ß pathway through Smad-Ski silencing [14] and in repression of neuronal genes in non-neuronal tissues [15],[16]. In the *Drosophila* system, HDAC1 controls segmentation genes through interaction with the Groucho co-repressor [17], executes Notch signalling readouts through interaction with CSL transcription factors [18], and HDAC1 has also been linked to silencing by Polycomb repressors [19],[20]. Thus, many endocrine, homeostatic, and developmental pathways employ HDACs in their gene control mechanisms.

**Figure 1:**
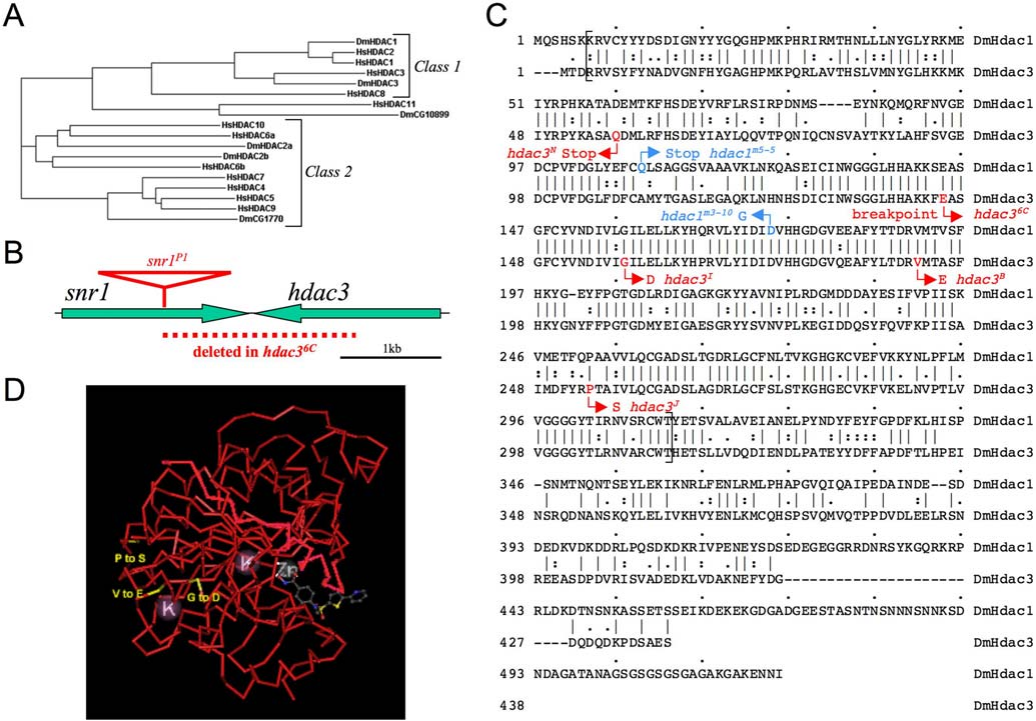
Loss-of-function mutations and family membership of *Drosophila* histone deacetylase-3 (HDAC3). (A) Phylogram showing relationships among the five *Drosophila* (Dm) and eleven human (Hs) histone deacetylases. The phylogram was generated from multiple sequence alignment of the catalytic domains (corresponding to amino acids 9–311 in *Drosophila* HDAC1) of each HDAC, using the ClustalW program (v. 1.83). The groupings of these HDACs into Class 1, Class 2, and a third HDAC11 class has been described [21]. (B) The genomic locus of fly *hdac3*. Arrows depict the convergent *hdac3* and *snr1* transcription units located at cytological locus 83A. The extent of the *hdac3^6C^* deletion, and the location of the P element insertion in *Snr1* used to generate this deletion by imprecise excision, are depicted. The extents of the transcription units are drawn to scale but, for sake of simplicity, coding versus non-coding and intronic regions are not distinguished. (C) Sequence alterations of *hdac* alleles. Amino acid sequences of *Drosophila* HDAC1 and HDAC3 are aligned with identical amino acids indicated by a line (|), highly similar amino acids by a colon (:) and similar amino acids by a period (.). Sequence alterations of the five *hdac3* mutations isolated here are shown in red and alterations in the two *hdac1* alleles used in Figure 2 are shown in blue. Brackets indicate approximate extents of the HDAC catalytic domain [21]. HDAC3 is about 50 residues shorter than HDAC1 at the C-terminus [59]. (D) Predicted locations of *hdac3* substitutions projected onto the HDAC three-dimensional structure. The *Drosophila* HDAC3 sequence was aligned to the human HDAC8 sequence, whose crystal structure has been solved [34]. The structural representation was generated using the Cn3D program (version 4.1) and the HDAC8 structural coordinates ([34], PDB ID code 1W22). Altered amino acids resulting from the three *hdac3* missense mutations were highlighted in the alignment, mapped onto the crystal structure, and are labelled in yellow. HDAC8 was crystallized as a deacetylase dimer [34] but, for clarity, only the monomer unit is shown. The catalytic pocket is located on the right surrounding the hydroxamic acid HDAC inhibitor, depicted in gray, which was complexed with HDAC8 in the crystal structure. Locations of bound zinc (Zn) and potassium (K) ions are also shown.

There are 11 HDAC family members in humans, defined by an approximately 350 amino acid homology region that encompasses the catalytic domain [21]. These have been classified into three major subfamilies, with class I containing HDACs 1, 2, 3 and 8, class II containing HDACs 4, 5, 6, 7, 9 and 10, and HDAC11 comprising a third distinct subtype [21] (see Figure 1A). In addition, the sirtuins represent yet another HDAC family, which are distinguished by their NAD-dependent reaction mechanism and are structurally unrelated to the family of 11 human HDACs. The large number of HDACs makes it difficult to determine which functions are shared and which can be uniquely assigned to individual family members. HDAC functional diversity is further complicated by the ability of HDACs to modify many protein substrates besides histones. Indeed, all three major HDAC subtypes are present in bacterial species [21], indicating that they likely evolved as protein deacetylases that only later acquired ability to act upon histones. In agreement with diversity of function, HDAC1 and 2 are largely nuclear, HDAC6 is cytoplasmic, and still other HDACs, including HDAC3, are found in both nucleus and cytoplasm [2], [22]–[25]. Within the nucleus, several transcription factors, including p53, GATA-1, and YY1, are HDAC substrates [26]–[28]. In the cytoplasmic compartment, tubulin deacetylation by HDAC6 has been described [24]. The large number of HDAC family members and the diversity of their protein substrates predict a myriad of HDAC regulatory functions in vivo.

HDAC functions are simpler to dissect in the *Drosophila* system, where there are only five HDAC family members [21] (Figure 1A). In addition, there are only two HDACs of the class I subtype: HDAC1 (also called DmRpd3) corresponding to the nuclear HDAC1/2 of mammals, and its most closely related fly paralog, HDAC3. Furthermore, HDAC1 and HDAC3 are the only two fly HDACs implicated in transcriptional control [29]. Genetic studies using HDAC1 mutations have identified roles in many processes including heterochromatin silencing, segmentation, and ecdysone receptor function [30]–[33]. However, the lack of HDAC3 mutations to date has impeded understanding of its biological functions in the fly system. Here we describe isolation of HDAC3 loss-of-function alleles and present phenotypic characterization. All five HDAC3 mutations are homozygous lethal at late larval or pupal stages. The mutant larvae have abnormally small imaginal discs, which we attribute to cell-autonomous induction of apoptosis rather than defects in cell proliferation.

## Results/Discussion

### Isolation of Recessive Lethal *Drosophila hdac3* Alleles

To investigate HDAC3 biological functions, we isolated a set of mutant alleles of fly *hdac3*. An initial *hdac3* allele was obtained by imprecise excision of a P transposon inserted into the neighboring gene, *snr1*, on the fly third chromosome (Figure 1B; see Methods). The excision yielded an approximately 1.8 kb deletion of genomic DNA that removes the 3′ two-thirds of both the *hdac3* and *snr1* coding regions. The deletion allele, termed *hdac3^6C^*, breaks at codon 145 of *hdac3*, within the HDAC catalytic domain. Since the severely truncated HDAC3 protein derived from this mutant allele, if stable, lacks critical portions of the catalytic domain [34],[35], it likely causes severe loss of HDAC3 function. However, the usefulness of this deletion allele for *hdac3* functional studies is limited by the simultaneous disruption of *snr1*.

To obtain mutations that specifically disrupt HDAC3, we next performed a noncomplementation screen for EMS-induced alleles that are lethal in trans to the *hdac3^6C^* deletion (see Methods). The recovery of *hdac3* alleles, as opposed to *snr1* alleles, was facilitated by use of a *P[snr1^+^]* rescue construct [36] in test crosses of the newly isolated lethal mutations (see Methods). We isolated four new *hdac3* mutations from this screen, each of which is lethal in trans to *hdac3^6C^*. All four *hdac3* mutant alleles cause lethality during pupal stages when either hemizygous or in transheterozygous combination with each other. Sequence analysis revealed that three of these alleles are missense mutations and one, *hdac3^N^*, converts Q57 to a stop codon (Figure 1C). Since this nonsense mutation eliminates the HDAC catalytic domain, it is likely a null allele. These results show that HDAC3 has unique functions required for fly development and viability, despite its 56% identity and 76% similarity to HDAC1 (Figure 1C).

The three-dimensional crystal structure of human HDAC8, a class I HDAC, has been determined [34],[35]. Based upon high primary sequence conservation, all class I HDACs likely exhibit similar overall protein folds. Thus, we projected our three *hdac3* substitutions onto the HDAC8 structure, to gain information about their possible effects on the protein (Figure 1D). The class I HDAC contains a single domain consisting of an 8-stranded parallel ß-sheet surrounded by 11 alpha-helices [34]. The catalytic site is located on one side and features a deep pocket, which is thought to bind the acetylated lysine substrate, and a Zn-binding site. All three missense substitutions are located on the opposite side, away from the catalytic site (Figure 1D). Thus, rather than specifically disrupting the catalytic center, these mutations may affect HDAC3 interactions with partner proteins, such as co-repressors, or they may cause structural destabilization. Two of these mutations affect residues that are highly conserved among HDACs; P254 is absolutely conserved among all 16 human and fly HDACs and V192 is conserved in all class I HDACs and in 14 of the 16 human and fly HDACs.

### *Hdac3* Mutations Suppress PEV but Show Little Effect on Polycomb Silencing

*Drosophila* HDAC1 has previously been implicated in two types of chromatin regulation: heterochromatin modification and gene silencing by the Polycomb system [19],[20],[32],[33]. Indeed, the first *Drosophila* HDAC mutation was isolated in HDAC1 based upon its modification of position-effect variegation (PEV) [33], a gene silencing event that occurs when a euchromatic gene is relocated to heterochromatin [37],[38]. In molecular terms, HDACs may promote heterochromatin silencing by deacetylating histone H3 on lysine 9, which renders this residue available for methylation by the heterochromatin silencing machinery [39],[40].

**Figure 2:**
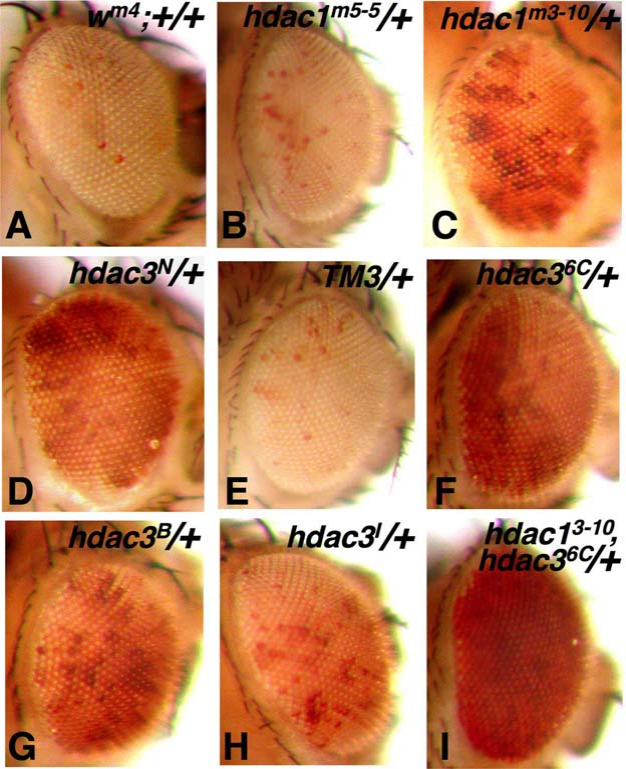
Suppression of position-effect variegation by *hdac* mutations. Panels show eye color phenotypes of adult males bearing the *In(1)w^m4^* X-chromosome plus wild-type third chromosomes (A) or heterozygous for the indicated *hdac* mutant alleles on the third chromosome (B–I). (B) and (C) show effects of *hdac1* alleles, (D) and (F–H) shows effects of *hdac3* alleles, and (I) shows robust suppression by an *hdac1 hdac3* double mutant. (E) shows that a *hdac^+^* third chromosome balancer (TM3) has no effect on PEV in this assay. The *hdac3* mutants used in these crosses were balanced using this version of TM3 and the fly in (E) was recovered as a sibling in the cross that yielded the fly in (D).

Heterochromatin silencing is conveniently assayed in *Drosophila* using *In(1)w^m4^*, an X-chromosome inversion that repositions the *white* eye color gene to heterochromatin [41]. This silences *white* expression in most cells of the eye, leading to a variegated eye color much lighter than normal (example in Figure 2A). Loss-of-function missense mutations in *Drosophila hdac1* are dominant suppressors of *w^m4^* variegation, whereas *hdac1* null mutations have little effect [32]. These results have been attributed to a poisoning effect of an expressed but inactive HDAC1 enzyme [32]. Similarly, we find that a null *hdac1* allele, Q109stop, has little effect on *w^m4^* variegation (Figure 2B) whereas a missense *hdac1* allele which alters a catalytic pocket residue, D174G, shows significant suppression (Figure 2C).

**Figure 3:**
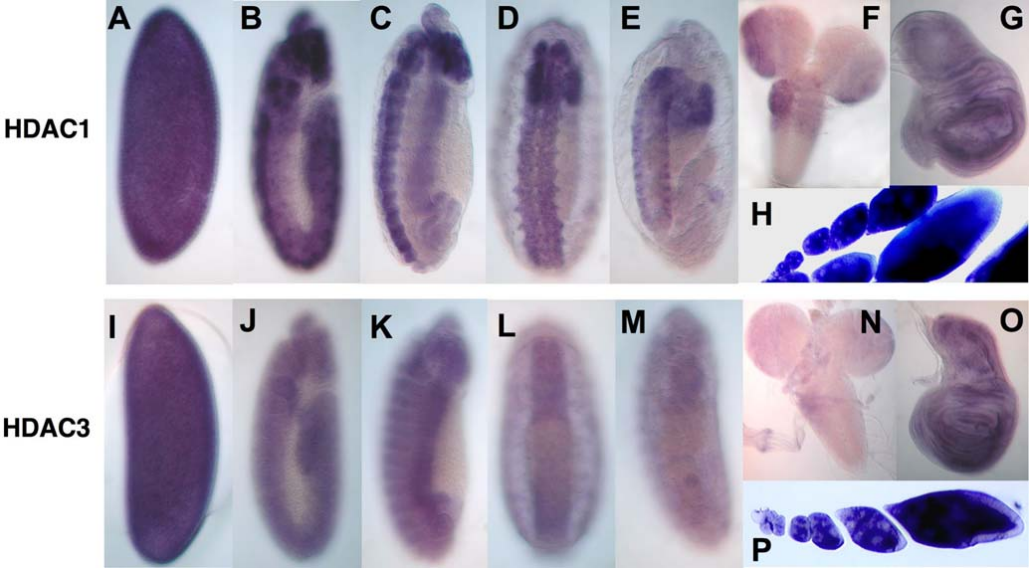
Comparisons of *hdac1* and *hdac3* mRNA expression during fly development. Panels show fly embryos and tissues after in situ hybridizations performed using *hdac1* (A–H) or *hdac3* (I–P) probes. Panels (A–E) and (I–M) show successive stages during embryogenesis, (F) and (N) show larval brain and central nervous systems, (G) and (O) show wing imaginal discs, and (H) and (P) show ovarioles with successive stages of oogenesis.

To investigate *hdac3* effects upon PEV, we tested each of our five *hdac3* mutations for *w^m4^* suppression. We found that heterozygosity for the *hdac3* null alleles, *hdac3^N^* and *hdac3^6C^*, led to strong suppression (Figure 2D and 2F) while the three missense alleles showed weaker effects, with moderate suppression by *hdac3^B^* and *hdac3^I^* (Figure 2G and 2H) and little or no effect with *hdac3^J^* (not shown). Thus, both *hdac3* and *hdac1* mutations can suppress PEV, indicating that both HDACs contribute to heterochromatin silencing. Indeed, an *hdac1*; *hdac3* double mutant shows more complete *w^m4^* suppression (Figure 2I) than any single mutant tested, consistent with HDAC1/HDAC3 functional overlap in heterochromatin. However, the suppressive effects of null mutations in the two genes are distinct, with *hdac3* nulls exerting robust dominant suppression and *hdac1* nulls displaying little effect. This might indicate that HDAC1 and HDAC3 affect heterochromatin by modifying distinct molecular targets. The distinct effects of the nulls could also reflect differences in accumulation levels of the two HDACs. If HDAC3 normally accumulates close to a critical threshold for function, then null mutations could suppress PEV through haploinsufficiency. In contrast, if HDAC1 normally accumulates in excess of the amount required, then null mutations would have little dominant effect and dominant suppression might require dominant-negative or antimorphic missense alleles, such as catalytic site substitutions. Although we have not compared HDAC1 and HDAC3 expression at the protein level, in situ hybridizations suggest that HDAC1 mRNA is more abundant than HDAC3 mRNA during fly development, at least in tissues such as the central nervous system (Figure 3).

*Drosophila* HDAC1 has also previously been implicated in gene silencing by the Polycomb system [19],[20]. The role of HDAC1 in Polycomb silencing is largely based upon its binding to the Polycomb silencing complex, PRC2 [20],[42], and enhancement of certain Polycomb group mutations by *hdac1* mutations [19]. However, *hdac1* single mutants have no discernible effect on expression of fly *Hox* genes [19], which are the most well-characterized targets of Polycomb silencing. This may reflect functional redundancy with other fly HDACs. Based upon sequence relatedness (Figure 1C), and their common roles in transcriptional control [29], HDAC3 is the best candidate to overlap with HDAC1 in Polycomb silencing. Thus, we investigated whether *Hox* gene silencing is altered in *hdac1*; *hdac3* double mutants. Immunostaining of double homozygous *hdac* null fly embryos revealed completely normal spatial patterns of the UBX and ABD-A Hox proteins (data not shown). In addition, enhancement of dominant Polycomb phenotypes was not observed in double heterozygotes bearing both Polycomb group and *hdac3* mutations. Thus, we were unable to detect a functional role for fly HDACs in Polycomb silencing. We note that the test on double mutant embryos is not definitive due to possible roles of maternally-provided *hdac* gene products.

### HDAC3 Mutants Have Small Imaginal Discs with Elevated Levels of Apoptosis

**Figure 4:**
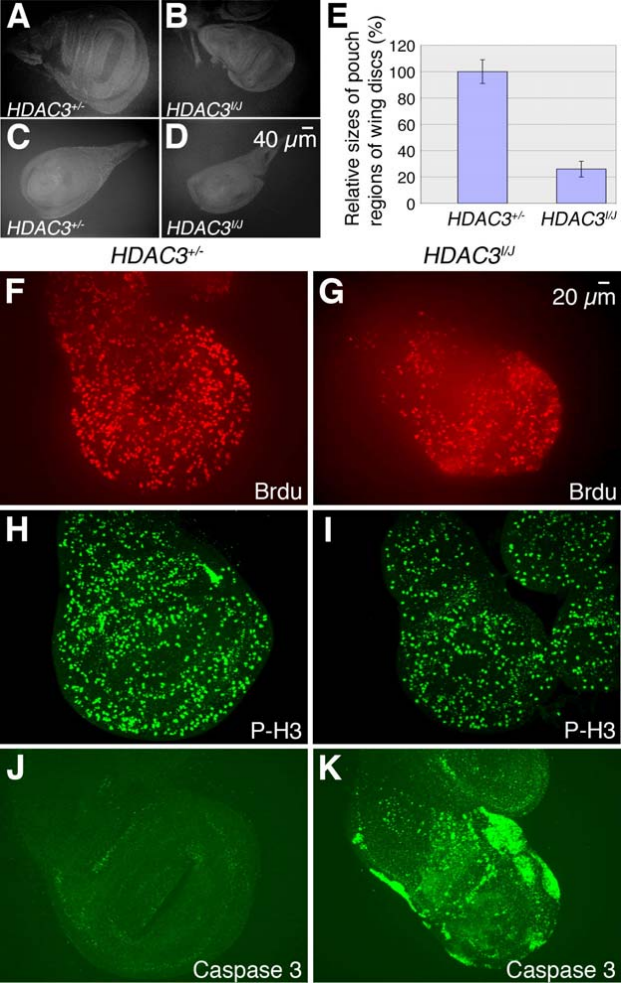
Reduced size in *hdac3* mutant wing discs is due to ectopic apoptosis. (A) Third instar larval wing imaginal disc from a *hdac3^+/−^* heterozygote stained with anti-Armadillo antibody and photographed at the same magnification as the wing disc from a *hdac3^I^/hdac3^J^* mutant larva shown in (B). (C) *hdac3^+/−^* leg disc. (D) *hdac3^I^/hdac3^J^* mutant leg disc. (E) Comparison of pouch region sizes of wing imaginal discs from *hdac3^+/−^* heterozygotes and *hdac3^I^/hdac3^J^* mutants. Eight individual wing discs of each genotype were stained with anti-Armadillo antibody, photographed, pouch regions were framed, and their two-dimensional areas were computed and analyzed by Student t-test. Error bars are standard deviation and p < 0.001. (F–K) show comparisons of *hdac3^+/−^* wing discs (F, H and J) to *hdac3^I^/hdac3^J^* mutant discs (G, I and K). (F and G) Discs were labelled and immunostained for bromodeoxyuridine (BrdU) to track cells transiting S phase. (H and I) Discs were immunostained with anti-phospho-histone H3 antibody to reveal mitotic cells. (J and K) Discs were immunostained with anti-activated caspase-3 antibody to reveal apoptotic cells. Very little apoptosis occurs in *hdac3^+/−^* heterozygous wing discs whereas robust ectopic apoptosis is seen in large portions of *hdac3^I^/hdac3^J^* mutant discs.

Although HDAC1 and HDAC3 appear to functionally overlap in regulating heterochromatin, HDAC3 clearly has a nonredundant role in development since all *hdac3* allelic combinations cause death during late third instar larval and early pupal stages. To investigate the cause of this recessive lethality, wandering third instar larvae bearing transheterozygous combinations of *hdac3* point mutations, or point mutations in trans to the small *hdac3^6C^* deficiency, were examined for developmental and tissue defects. We found that imaginal discs in these mutants are significantly reduced in size compared to wild-type (Figure 4A–4D). The pouch region of the wing disc appears particularly small and quantitative measurements revealed reductions to about 30% of normal size (Figure 4E). The small disc phenotype was also observed in *hdac3^6C^* homozygotes bearing a *P[snr1^+^]* transgene to cover *snr1* loss.

The small disc phenotype could result from decreased proliferation or increased levels of apoptosis during development. To assess proliferation, discs from *hdac3* mutant larvae were immunostained with anti-BrdU to detect DNA replication and with anti-phospho-histone H3 to detect mitotic cells. Although the discs are smaller than wild-type, the relative densities of signals with these S- and M-phase markers did not appear significantly altered (Figure 4F–4I). To track apoptosis, *hdac3* mutant discs were immunostained with antibody against activated caspase-3. In contrast to wild-type wing discs, which show little apoptosis (Figure 4J), *hdac3* mutant wing discs display robust and widespread apoptosis (Figure 4K). In general, apoptosis in the *hdac3* mutants is more prominent in the wing blade versus the notal portion of the disc and the precise patterns of apoptotic cells can vary considerably from disc to disc ( Figure S1A and S1B). Thus, the main cause of reduced imaginal disc size in *hdac3* mutants is abnormally elevated apoptosis rather than reduced cell proliferation.

### HDAC3 Loss Induces *Hid* Expression and Apoptosis through a Cell-Autonomous Mechanism

**Figure 5:**
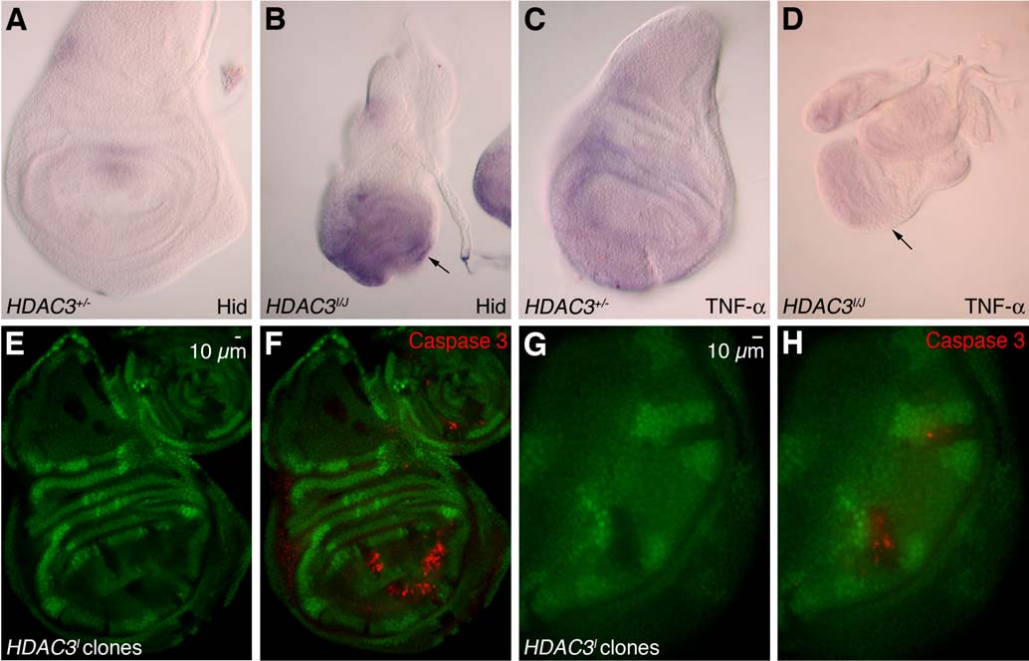
Induction of apoptosis in *hdac3* mutant wing discs is cell-autonomous and accompanied by *hid* mRNA induction. (A–D) show in situ hybridizations to track the indicated mRNAs in imaginal discs from *hdac3^+/−^* heterozygotes (A and C) versus from *hdac3^I^/hdac3^J^* mutants (B and D). Hybridization probes detect *hid* mRNA (A and B) or *TNF-*α mRNA (C and D). (E–H) *hdac3^I^* homozygous mutant clones were generated in a field of *hdac3^+^*/*hdac3^I^* heterozygous wing disc tissue. *hdac3^I^* mutant clones are GFP-negative. (F) Apoptotic cells detected with anti-activated caspase-3 antibody (red) are confined to *hdac3^I^* homozygous mutant tissue. The green channel of the optic section (F) is shown alone in (E) to display GFP-negative regions. (G and H) Examples of *hdac3^I^* mutant clones at higher magnification.

The *Drosophila* cell death regulatory pathway contains numerous components that are conserved in mammalian systems ([43],[44] for reviews). These include initiator and effector caspases, inhibitor of apoptosis proteins (IAPs) which bind caspases, and pro-apoptotic proteins which interfere with IAP function. In the fly pathway, control is frequently exerted at the level of pro-apoptotic protein expression, where up-regulation of factors such as HID, RPR and GRIM promotes caspase-dependent cell death [45]. These correspond to SMAC/DIABLO regulatory factor in mammals [46]. To determine if HDAC3 loss leads to caspase activation via this conserved pathway, we performed in situ hybridizations to track levels of *hid* mRNA. As shown in Figure 5A and 5B, there is a dramatic increase in *hid* mRNA accumulation in *hdac3* mutant wing discs compared to wild-type.

In addition to these pro-apoptotic proteins, there are also cell-extrinsic cues that influence apoptosis through death receptor signaling. For example, tumor necrosis factor-alpha (TNF-α) signaling is implicated in apoptotic control in both flies and mammals [43],[47]. To address whether cell-extrinsic mechanisms are involved in apoptotic induction in *hdac3* mutants, we first performed in situ hybridization to assess levels of TNF-α *(eiger)* mRNA, which revealed no significant change (Figure 5C and 5D). Second, we generated somatic clones of *hdac3* mutant cells within imaginal discs and immunostained these discs for activated caspase. The tight correspondence between territories of *hdac3* mutant cells, as visualized by GFP-negative regions, and regions of caspase activation (Figure 5E–5H) indicates that HDAC3 loss triggers apoptosis through a cell-autonomous mechanism. Taken together, these results suggest that HDAC3 impacts the cell-intrinsic apoptotic control pathway at a position upstream of *hid* mRNA accumulation. Further studies will be needed to identify the precise protein substrate(s) targeted by HDAC3 in this pathway. One possibility is that *hid* transcription is up-regulated through hyper-acetylation of histones associated with *hid* gene chromatin. Alternatively, there may be non-histone proteins targeted by HDAC3 whose acetylation state can regulate the apoptotic pathway. The observation that fly and vertebrate HDAC3 are found in both the nucleus and cytoplasm [22],[23],[25] is consistent with either possibility.

### Analysis of Apoptosis and Proliferation in HDAC1 Mutant Wing Discs

To compare effects of *hdac3* versus *hdac1* loss-of-function in imaginal discs, we analyzed disc size, apoptosis, and proliferation in wing discs from *hdac1* mutant larvae. Initially, we sought to analyze whole wing discs from the severe loss-of-function *hdac1* mutants, *m3-10* and *m5-5*. However, these homozygous mutants, and the *m3-10/m5-5* transheterozygote, failed to survive beyond the end of the second larval instar, precluding analysis of mature larvae. To circumvent this problem, we pursued two approaches: 1) we analyzed mosaic wing discs bearing *m3-10* mutant clones and 2) we used a hypomorphic *hdac1* allele, *l(3)04556*, which contains a P element insertion into the 5′-UTR [30],[32].

**Figure 6:**
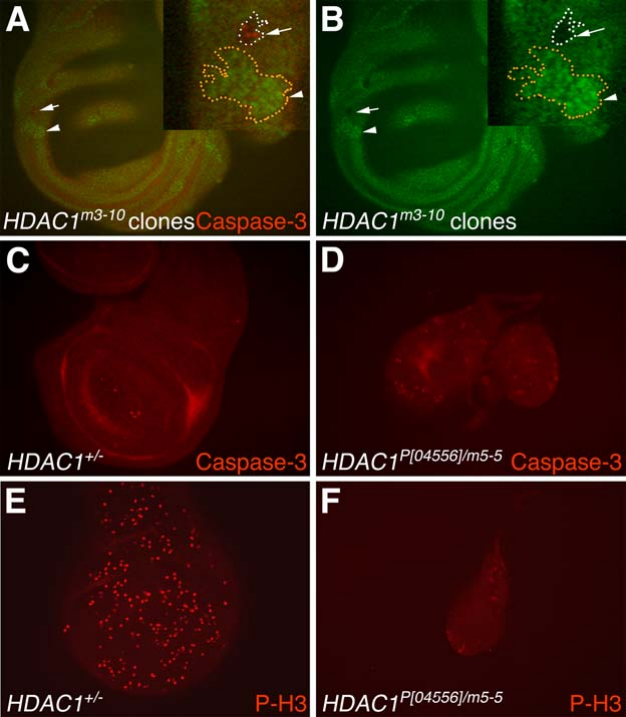
Analysis of *hdac1* mutant wing discs and clones for effects upon apoptosis and proliferation. (A,B) *hdac1^m3-10^* homozygous mutant clones were generated in a field of *hdac1^+^/hdac1^m3-10^* heterozygous wing disc tissue. (A) Wing disc showing a GFP-negative *hdac1^m3-10^* mutant clone (arrow) and its wild- type twinspot clone marked by strong GFP expression (arrowhead). These clones are highlighted at higher magnification in the inset (upper right corner). A subset of cells within the *hdac1^m3-10^* mutant clone (arrow) stain with anti-activated caspase-3 antibody (red). This mutant clone is much smaller than its wild-type twinspot clone (arrowhead). (B) The green channel images of the optic sections in (A) are shown alone in (B) to better display GFP-negative regions. Large central regions of the disc that appear unstained are out of the focal plane. (C) Control wing disc from a heterozygous *hdac1^+/−^* late wandering third instar larva stained with anti-activated caspase-3 antibody shows very few apoptotic cells. (D) *hdac1^P[04556]/m5-5^* mutant wing disc (left) and haltere disc (right) showing scattered apoptotic cells (red) stained with anti-activated caspase-3 antibody. (E,F) Wing discs were immunostained with anti-phospho-histone H3 antibody (red) to reveal mitotic cells. The density of mitotic cells in the heterozygous *hdac1^+/−^* disc (E) is greater than in the *hdac1^P[04556]/m5-5^* disc (F). Panels C–F also illustrate that *hdac1* mutant wing discs are greatly reduced in size compared to control wing discs. All images besides the insets in A,B were taken at the same magnification.

As shown in Figure 6A and 6B, *hdac1^m3-10^* wing disc clones are significantly smaller than the *hdac3* clones described above (Figure 5). In the example outlined at higher magnification (Figure 6A), several features are illustrated. First, the *m3-10* homozygous mutant clone is much smaller than its wild-type twinspot. Second, a subset of cells in the clone stain with anti-activated caspase-3, indicating some degree of apoptotic induction. However, numerous *m3-10* clones of similar size which lacked detectable caspase-3 were also observed. This variability, together with the dramatic difference in *hdac1* versus *hdac3* clone sizes, suggests that apoptosis alone is unlikely to account for the small sizes of *hdac1* clones. Since previous studies have shown that *hdac1* knockdown impairs cell cycle progression in cultured fly cells [29], we suspected that the observed *hdac1* clone sizes may result primarily from defects in proliferation rather than abnormal apoptosis.

To further address these possibilities, we analyzed whole discs isolated from a strong hypomorphic allelic combination, *hdac1^P[04556]^/hdac1^m5-5^*. These transheterozygous larvae live until pupal stages. Figure 6C–6F illustrates that wing discs from this *hdac1* strong hypomorph are dramatically reduced in size compared to wild-type. These *hdac1* discs are also smaller than the *hdac3* mutant discs described above (Figure 4). When stained for activated caspase-3, this *hdac1* mutant shows sporadic isolated cells and small clusters of cells undergoing apoptosis (Figure 6D). This limited distribution differs from the robust and widespread apoptosis seen in *hdac3* mutant discs (Figure 4K and Figure S1A and S1B). When the *hdac1* mutant is stained for a mitotic marker (Figure 6F), fewer mitotic cells are detected at a reduced density compared to heterozygous control discs (Figure 6E). Taken together, the results suggest that *hdac1* mutant imaginal discs are reduced in size primarily because of a proliferation defect whereas the major defect in *hdac3* discs is ectopic apoptosis.

### HDAC Inhibition and Apoptotic Control in Cancer

There is significant interest in deciphering the molecular consequences of HDAC loss because HDAC chemical inhibitors show potential as anticancer agents in preclinical studies with cell and animal models [48],[49]. Consistent with *hdac3* genetic loss-of-function described here, chemical HDAC inhibitors induce tumor cell killing through robust activation of apoptosis [50],[51]. Remarkably, HDAC inhibitors promote apoptotic cell death selectively in tumor cells versus in non-transformed cells [50]. However, the molecular mechanisms that connect HDAC inhibition to mammalian cell death are not yet clear. Previous studies have described up-regulation of cell-extrinsic death receptor pathways as well as effects upon components in intrinsic pathways that operate through mitochondria to trigger apoptosis [50]. Although the sum of the evidence suggests multiple inputs, the cell autonomy of the response in the fly system (Figure 5) suggests that cell-intrinsic regulation of pro-apoptotic factors makes a key contribution. Besides pro-apoptotic factors such as hid/Smac/Diablo, the Bcl2 class of cell-intrinsic regulators are commonly used in the mammalian pathway [52]. However, relatively little is known about regulatory mechanisms of the Bcl2 factors Buffy and Debcl in *Drosophila* [44].

It is also currently unclear which of the 11 human zinc-dependent HDACs must be inhibited to trigger tumor cell apoptosis. Many of the commonly used HDAC inhibitors such as trichostatin (TSA) are broad spectrum antagonists that affect all family members [50]. Indeed, encouraging preclinical results have been obtained with suberoylanilide hydroxamic acid, which affects all eleven human HDACs, and with valproic acid, which inhibits eight of the eleven including all class I enzymes [48]–[50], [53]–[55]. Our data show that loss of HDAC3 alone is sufficient to trigger robust apoptosis in fly tissues, indicating that this conserved HDAC plays a substantial role among family members in apoptotic control. Further studies will be needed to assess if specific inhibition of human HDAC3 makes a critical contribution to tumor cell killing.

### Division of Labor and Functional Roles of HDAC1 and HDAC3

A recent transcription profile microarray study using cultured fly cells suggests that HDAC1 and HDAC3 are the only two fly HDACs with major functions in transcriptional control [29]. Thus, HDAC3, the closest fly paralog by sequence, is also likely to be the most functionally related fly family member to HDAC1. Our isolation of *hdac3* mutations provided the opportunity to begin to assess this HDAC1/HDAC3 relationship in vivo. Our results show that both *hdac3* and *hdac1* mutations can suppress PEV (Figure 2), indicating roles for both HDACs in heterochromatin regulation. The fact that an *hdac1*; *hdac3* double mutant displays an enhanced effect (Figure 2I) suggests that both HDACs make significant contributions to this process. The simplest molecular explanation is that both enzymes directly deacetylate histone residues that then become methylated in heterochromatin. However, RNA interference experiments using cultured fly S2 cells suggest that HDAC1 is the predominant histone-modifying enzyme, with little unique contribution detected from HDAC3, at least in this cell type [29]. Thus, HDAC3 function at heterochromatin could reflect deacetylation of either histone or non-histone substrates.

In a developmental context, requirements for HDAC3 function distinct from HDAC1 could occur at times or in cell types that accumulate HDAC3 but not HDAC1. However, the spatial distributions of *hdac3* and *hdac1* mRNAs are both widespread (Figure 3), their temporal profiles during development are similar [25], and we have not detected individual tissues or cell types where *hdac3* product accumulates without *hdac1*. In general, *hdac1* mRNA levels appear more abundant than those of *hdac3* especially in the CNS (Figure 3). This is consistent with the report that the genome-wide transcriptional response to HDAC1 knockdown in cultured fly cells is more robust and involves a larger number of affected genes as compared to HDAC3 knockdown [29]. Thus, HDAC1 may be needed for certain processes that do not require HDAC3. Indeed, both our in vivo results (Figure 6), and fly S2 cell studies [29], support a preferential role for HDAC1, as opposed to HDAC3, in controlling cell proliferation.

The most significant HDAC3 function revealed by our genetic approach is in control of cell death (Figures 4 and 5). Since HDAC3 loss is by itself sufficient to trigger ectopic apoptosis, neither HDAC1 nor other fly HDACs can substitute for this requirement, at least in imaginal disc tissue. These results contrast with findings on apoptosis from an HDAC knockdown study using cultured fly cells. Although treatment with a broad-specificity HDAC inhibitor, trichostatin, did induce apoptosis in S2 cells, neither HDAC1 nor HDAC3 knockdown, nor the double knockdown, affected cell viability [29]. One possible explanation for this discrepancy is that the degree of *hdac3* loss-of-function produced by our mutations in vivo is more severe than the degree achieved by RNA interference. Alternatively, the conflicting results may reflect tissue differences in the response to HDAC loss; S2 cells are derived from embryonic neuronal cells whereas the most dramatic induction of apoptosis is seen in a larval epithelial tissue, imaginal discs. Indeed, we note that there is little apoptotic induction in nervous system tissue of the same larvae that display robust induction in discs (Figure S1). Further studies will be needed to determine the mechanisms and pathways by which HDACs control apoptosis in various tissues during normal development as well as in mammalian cell and animal models for cancer.

## Materials and Methods

### Isolation and Characterization of *Hdac3* Mutant Alleles

The *hdac3^6C^* deletion was generated by imprecise excision of a P element, *P[PZ ry+]Snr1^01319^*, inserted into the neighboring *snr1* gene. After introduction of a P transposase source, potential local deficiencies were recovered as *ry^−^* progeny that failed to complement lethality of the original P insertion. PCR analysis of genomic DNA from 23 independent balanced lethal lines, using *snr1* and *hdac3* primers, identified a single line bearing an approximately 1.8 kb local deletion. Gel-isolation and sequencing of the altered PCR product defined a deletion that removed about two-thirds of the *hdac3* coding region (Figure 1C) plus all *snr1* DNA 3′ to the original P insertion.

The four *hdac3* point mutations were isolated based upon failure to complement recessive lethality of *hdac3^6C^*. Homozygous males of genotype *P(ry^+t7.2^ = neoFRT)82B ry^506^* were treated with EMS as described [56] and mutagenized third chromosomes were balanced over *TM6B*, *Tb Sb*. Males bearing the mutagenized chromosomes were mated to females of genotype *w^−^*; *hdac3^6C^ P[Snr1^+^ w^+^]/TM6B,Tb* and progeny were scored for absence of viable non-Tubby pupae. The presence of the *P[Snr1^+^]* rescue construct [36], kindly provided by Andrew Dingwall and recombined onto the *hdac3^6C^* chromosome, facilitated recovery of *hdac3* lethal mutations. Four lethal mutations were recovered from 5000 matings, siblings were used to establish balanced lethal stocks, and test crosses confirmed that each mutation is lethal in trans to *hdac3^6C^*. The four mutant lesions were identified by PCR sequencing of genomic DNA isolated from adult flies heterozygous for each mutation.

### Tests for Suppression of Position Effect Variegation

To test the four EMS-induced *hdac3* alleles, females of genotype *In(1)w^m4^/In(1)w^m4^*; *P(ry^+t7.2^ = neoFRT)82B ry^506^/P(ry^+t7.2^ = neoFRT)82B ry^506^* were mated to males of genotype *P(ry^+t7.2^ = neoFRT)82B ry^506^ hdac3/TM3*, *Sb* and the non-Stubble male progeny were scored for eye color phenotypes. These progeny are heterozygous for the *hdac3* allele and essentially isogenic for the remainder of the third chromosome to minimize background effects. Additional *hdac* mutants were tested by mating *In(1)w^m4^*; *FRT82B* females to males of genotype *hdac1^m5-5^/TM3*, *hdac1^m3-10^/TM3*, *hdac3^6C^/TM3* or *hdac1^m3-10^ hdac3^6C^/TM3*. The latter stock contains a recombinant third chromosome bearing mutations in both *hdac1* and *hdac3*. These *hdac1* alleles were isolated previously as members of complementation group *l(3)64Cc* in a deficiency non-complementation screen [57].

### Generation of *Hdac3* and *Hdac1* Somatic Clones

For *hdac3* clones, flies of genotype *FRT82B hdac3^I^/TM6B*, *Tb* or *FRT82B hdac3^J^/ TM6B*, *Tb* were crossed to *yw hsFlp*; *FRT82B ubi-GFP* flies at 25°C. For *hdac1* clones, flies of genotype *FRT2A hdac1^m3-10^/TM6B*, *Tb* were crossed to *hsFlp*; *FRT2A ubi-GFP* flies. Progeny of these crosses were heat shocked as first instar larvae at 37°C for 1.5 hours to induce expression of FLP recombinase and mitotic recombination. Larvae were then reared at 25°C for 72 hours prior to dissection and immunostaining. Somatic clones homozygous for the *hdac* mutations were detected as regions lacking GFP expression.

### Immunostaining of Larval Tissues

Wandering third instar larvae were dissected in PBS (137 mM NaCl, 2.7 mM KCl, 4.3 mM Na_2_HPO_4_ and 1.4 mM KH_2_PO_4_, pH 7.4), and tissues were fixed with 3.7% formaldehyde in PBS for one hour at room temperature and then washed three times in PBS plus 0.1% Triton X-100 (PBT). Mouse anti-armadillo and mouse anti-BrdU antibodies were obtained from the Developmental Studies Hybridoma Bank (University of Iowa) and were used at 1/100 dilution. Rabbit anti-phosphorylated histone H3 (pSer10, Sigma) was used at 1/400 dilution. Rabbit anti-activated caspase-3 antibody was obtained from Idun Pharmaceuticals and used at 1/2000 dilution. Primary antibody incubations were performed overnight at 4°C after dilution into PBT. Anti-rabbit and anti-mouse fluorescent conjugated secondary antibodies (Molecular Probes) were diluted 1/200 in PBT and incubated with tissue samples at room temperature for 2 hours. Confocal images were taken with a Zeiss Axioplan2 confocal microscope.

### BrdU Incorporation

Larval central nervous systems and imaginal discs were dissected in PBS, transferred to M3 complete medium containing 0.4 mg/ml BrdU (Roche), and incubated at 25°C for 30 minutes to allow incorporation. Tissues were then fixed as above for immunostaining, incubated in 2N HCl for 30 minutes at room temperature, and then washed three times in PBT before addition of anti-BrdU monoclonal antibody.

### In Situ Hybridization

Antisense probes were prepared by in vitro transcription with digoxygenin uridine triphosphate using linearized plasmids bearing the relevant cDNA inserts as template. The *hid, TNF-*α and *hdac3* probes were generated using cDNAs obtained from the Berkeley Drosophila Genome Project (http://www.fruitfly.org/DGC/) and the *hdac1* probe was derived from a full-length cDNA clone, pBS-Rpd3, obtained from Pierre Spierer. Sense probes of similar lengths, which were prepared and hybridized in parallel, yielded no signals above background. Whole mount in situ hybridizations and signal detection were performed as described [58].

## Supporting Information

Figure S1 Apoptotic induction in hdac3 mutant larval tissues. All panels show tissues immunostained with anti-activated caspase-3 antibody. (A–B) Examples of wing imaginal discs from hdac3I/hdac3J mutants to illustrate variable patterns of apoptosis. Apoptotic cells occur more frequently in the wing blade region than in the portion that will give rise to notum. (C and D) Portions of brain lobe (br), eye (ed), and antennal (ad) imaginal discs from hdac3+/− heterozygous (C) and hdac3I/hdac3J mutant (D) larvae. Very few cells are labeled either in developing brain lobes (br) or eye and antenna imaginal discs of hdac3+/− heterozygous third instar larvae. Apoptotic induction in the hdac3 mutant is seen in portions of the eye and antennal discs (arrows and arrow heads) but not in the brain lobes. (E and F) Haltere (hd) and leg (ld) imaginal discs from hdac3+/− heterozygous (E) and hdac3I/hdac3J mutant (F) larvae. Very few apoptotic cells are seen in control discs from heterozygotes whereas robust apoptosis is evident throughout the leg disc (arrows) from the hdac3 mutant.
(5.18 MB TIF)
