# Supplementary figures and images for: Drosophila Histone Deacetylase-3 Controls Imaginal Disc Size through Suppression of Apoptosis
Source: PLoS Genet. 2008 Feb 29;4(2):e1000009. doi: 10.1371/journal.pgen.1000009 (PMC2265479; doi:10.1371/journal.pgen.1000009)

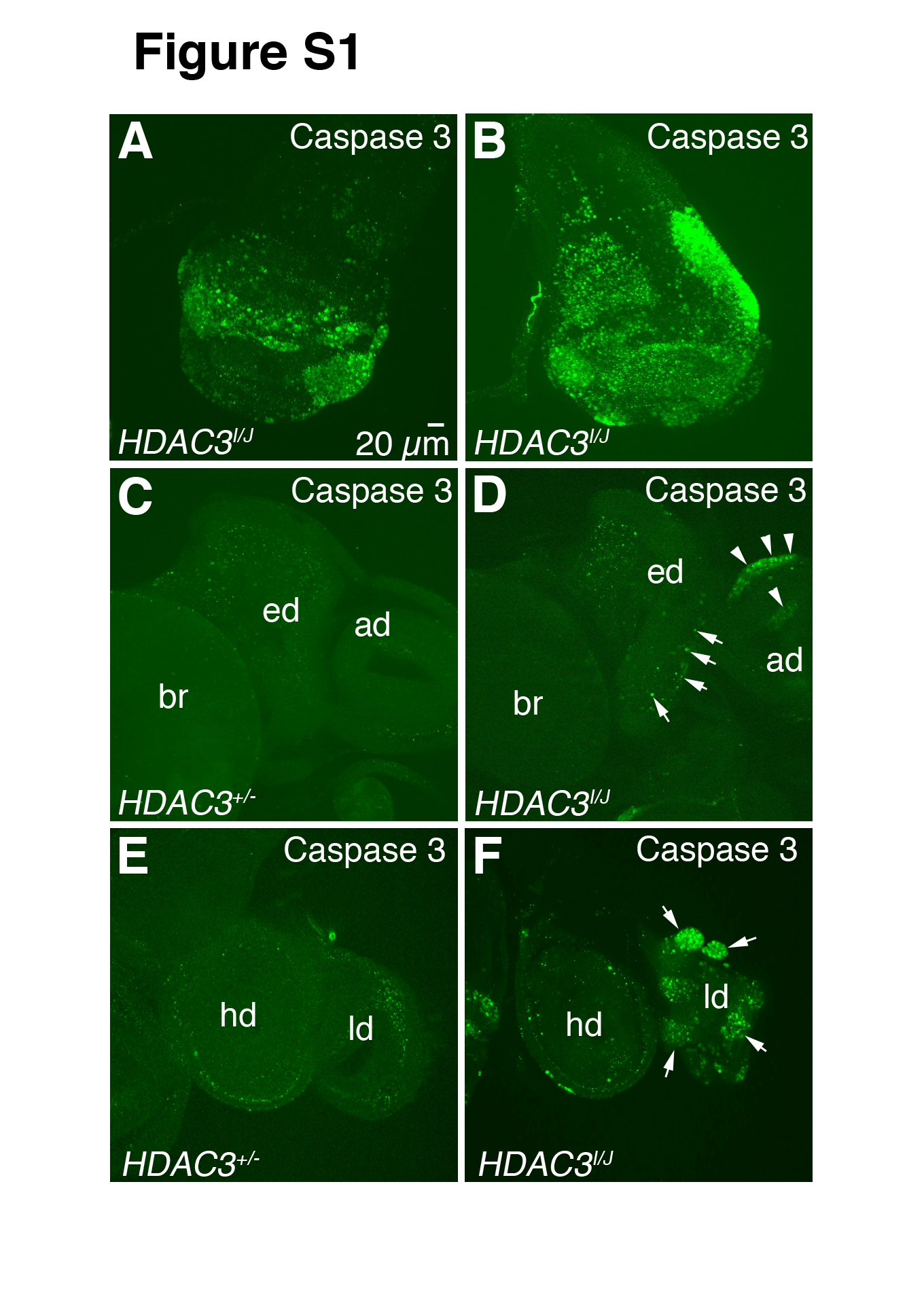

Supplement: Figure S1 — Apoptotic induction in hdac3 mutant larval tissues. All panels show tissues immunostained with anti-activated caspase-3 antibody. (A–B) Examples of wing imaginal discs from hdac3I/hdac3J mutants to illustrate variable patterns of apoptosis. Apoptotic cells occur more frequently in the wing blade region than in the portion that will give rise to notum. (C and D) Portions of brain lobe (br), eye (ed), and antennal (ad) imaginal discs from hdac3+/− heterozygous (C) and hdac3I/hdac3J mutant (D) larvae. Very few cells are labeled either in developing brain lobes (br) or eye and antenna imaginal discs of hdac3+/− heterozygous third instar larvae. Apoptotic induction in the hdac3 mutant is seen in portions of the eye and antennal discs (arrows and arrow heads) but not in the brain lobes. (E and F) Haltere (hd) and leg (ld) imaginal discs from hdac3+/− heterozygous (E) and hdac3I/hdac3J mutant (F) larvae. Very few apoptotic cells are seen in control discs from heterozygotes whereas robust apoptosis is evident throughout the leg disc (arrows) from the hdac3 mutant. (5.18 MB TIF) [file pgen.1000009.s001.tif]
